# Unmasking the Giant Condyloma: A Case Report and Literature Review of Buschke-Löwenstein Tumor

**DOI:** 10.7759/cureus.98967

**Published:** 2025-12-11

**Authors:** Vasileios Tzikoulis, Anastasios Anastasiadis, Dimitrios Memmos, Stavros Tsiakaras, Ioannis Mykoniatis, Fotios Dimitriadis, Konstantinos Papathanasiou, Christos Roidos, Loukas Charalambous, Georgios Gousis, Nikolaos Tserkezis, Maria Kougioni, Dimitrios Oikonomou, Aimilios Lallas, Lavrentis Papalavrentios, Georgios Dimitriadis

**Affiliations:** 1 First Department of Urology, School of Medicine, Aristotle University of Thessaloniki, “G. Gennimatas” General Hospital, Thessaloniki, GRC; 2 School of Medicine, Aristotle University of Thessaloniki, Thessaloniki, GRC; 3 Plastic Surgery, Oikonomouclinic, Thessaloniki, GRC; 4 First Department of Dermatology, School of Medicine, Aristotle University of Thessaloniki, Thessaloniki, GRC; 5 Fourth Department of Internal Medicine, School of Medicine, Aristotle University of Thessaloniki, Thessaloniki, GRC

**Keywords:** buschke-löwenstein tumor, genital warts, giant condyloma acuminatum, human papillomavirus, perineal lesions

## Abstract

Buschke-Lowenstein tumors (BLTs) are rare, slow-growing, cauliflower-like lesions of the perineal region. Despite its benign histology, it may be a precursor of squamous cell carcinoma (SCC), as it possesses the potential for malignant transformation. Multiple sexual partners and early sexual debut are among the main risk factors. Topical therapy and minimally invasive excisional therapies may be applied in mild cases. Radiotherapy, chemotherapy, and immunotherapy have also been described, presenting limited efficacy. Surgery remains the gold-standard treatment, especially for extensive lesions, as demonstrated in our case. We report the case of a heterosexual 33-year-old male patient with extensive penoscrotal, pubic, inguinal, and perineal anodyne lesions, ongoing for 14 months. In the past two years, two different sexual partners were reported. Due to the extent of the lesions, a multidisciplinary approach was required, consisting of a urologist and a plastic surgeon. The histopathological analysis confirmed the original diagnosis of a BLT consistent with human papillomavirus effect, ruling out any possibility of malignant transformation to SCC. Skin flaps were used to reconstruct the skin defect. A complete wound closure was achieved at four weeks postoperatively. A year later, the patient is free of any similar lesions. Surgical excision of the tumor is still the benchmark of BLT treatment. It features the ability to confirm the diagnosis as well. Based on the extent of the lesions and the consequent skin defect, a multidisciplinary approach is strongly recommended. Due to the high recurrence rate and potential malignant transformation, a close follow-up is necessary.

## Introduction

Buschke-Löwenstein tumor (BLT), also known as giant condyloma acuminatum (GCA), is a rare, slow-growing, cauliflower-like tumor of the anogenital region that exhibits locally destructive and infiltrative behavior. It is strongly associated with human papillomavirus (HPV) subtypes 6 and 11, which are the causative agents of hyperplastic epithelial lesions, and more rarely with HPV subtypes 16 and 18, which are responsible for the notable tendency toward malignant transformation, typically resulting in squamous cell carcinoma (SCC) [1,2]. These tumors lack lymphovascular or neural invasion, and distant metastasis is not reported. It is considered that BLT is midway between GCA and SCC, and its malignant transformation risk accounts for 40-60% of the patients [3,4].

BLT was first described in 1925 by Abraham Buschke and Ludwig Löwenstein [5]. Since then, only a few sporadic case reports of BLT have been published, due to its very low incidence, which is estimated to be 0.1% of the sexually active population. BLT affects 2.7 times more of the male population, and the mean age at diagnosis is 43.9 years. It is regarded that almost everyone will present at least one HPV infection during their lifetime, usually asymptomatic and spontaneously resolved. Overall, 2% of these patients will progress to condyloma acuminata [6].

The transmission of this infection occurs through sexual intercourse and even by skin-to-skin contact. Multiple sexual partners, early sexual debut, sexually transmitted diseases (STDs), male homosexuality, poor hygiene, and immunocompromised status constitute the main risk factors [7].

The diagnosis of BLT is based on the patient’s history and a thorough clinical examination. An exophytic, cauliflower-like, white-yellowish tumor that may invade and/or destroy the surrounding tissues is usually seen. Typically, the lesions are located on the penis and vulva in approximately 90% of cases. Rarely, the anorectal region and/or the urethra are involved. Most commonly, the disease is observed in immunocompromised patients [8,9]. The use of imaging methods such as MRI and CT can be useful in staging and surgical planning of the disease, while histological examination is necessary in ruling out malignant transformation to SCC [10].

This article was previously presented as a meeting abstract at the 2025 ESGURS-ESAU Joint Meeting in Turin, on October 2, 2025.

## Case presentation

A 33-year-old male presented to the urology department complaining of painless lesions on the external genitalia, ongoing for 14 months. He had no significant past medical history, was heterosexual, and was employed as a software developer. In the past two years, he had two different sexual partners, without a known medical history.

**Figure 1:**
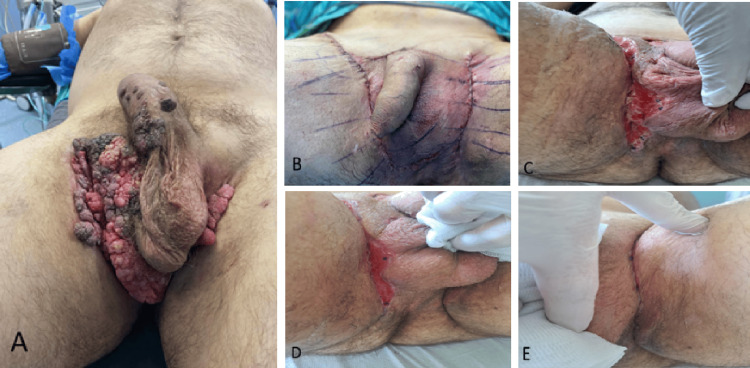
Pre- and postoperative images. (A) Preoperative image of the lesions. (B) Perioperative image, depicting the use of flaps. (C, D) Wound healing in one and two weeks postoperatively. (E) Complete wound healing four weeks postoperatively.

Extensive exophytic lesions resembling condylomata were observed on the ventral surface of the penis, the right hemiscrotum, bilaterally in the inguinal regions, and in the perineal area. Enlarged, palpable lymph nodes in the right inguinal region were noted. No ulcerations or other pathological signs were detected. The urethra and anus were free of lesions (Figure 1A).

**Figure 2:**
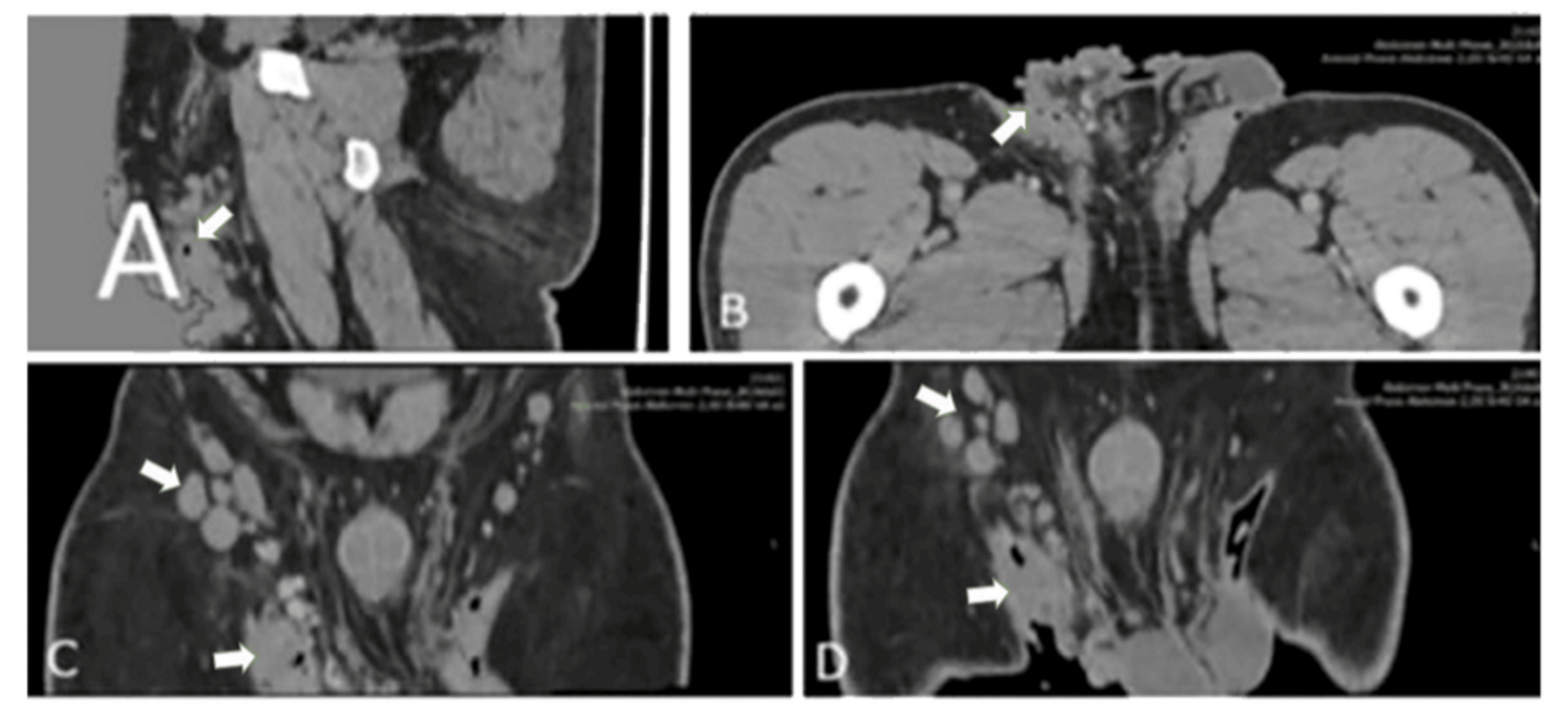
CT images. (A) Sagittal image. (B) Traverse image. (C) Coronal image. (D) Coronal image. Significant irregular thickening with lobulated margins in the perineum, as well as in the scrotum, more prominently on the right, with multiple enlarged lymph nodes.

The CT scan revealed an extensive plaque-like skin process in the anatomical region of the perineum, as well as in the scrotum. In the right inguinal region, multiple lymph nodes were observed, measuring up to approximately 1.5 × 2.5 cm. Despite the displacement of the right external iliac and common femoral vessels, a pathological lymph node enlargement was identified in the same region, with approximate dimensions of 1.8 × 3.4 cm. Additionally, a lymph node measuring 0.5 × 1 cm was noted adjacent to the peripheral portion of the right external iliac vessels (Figures 2A-2D).

**Figure 3:**
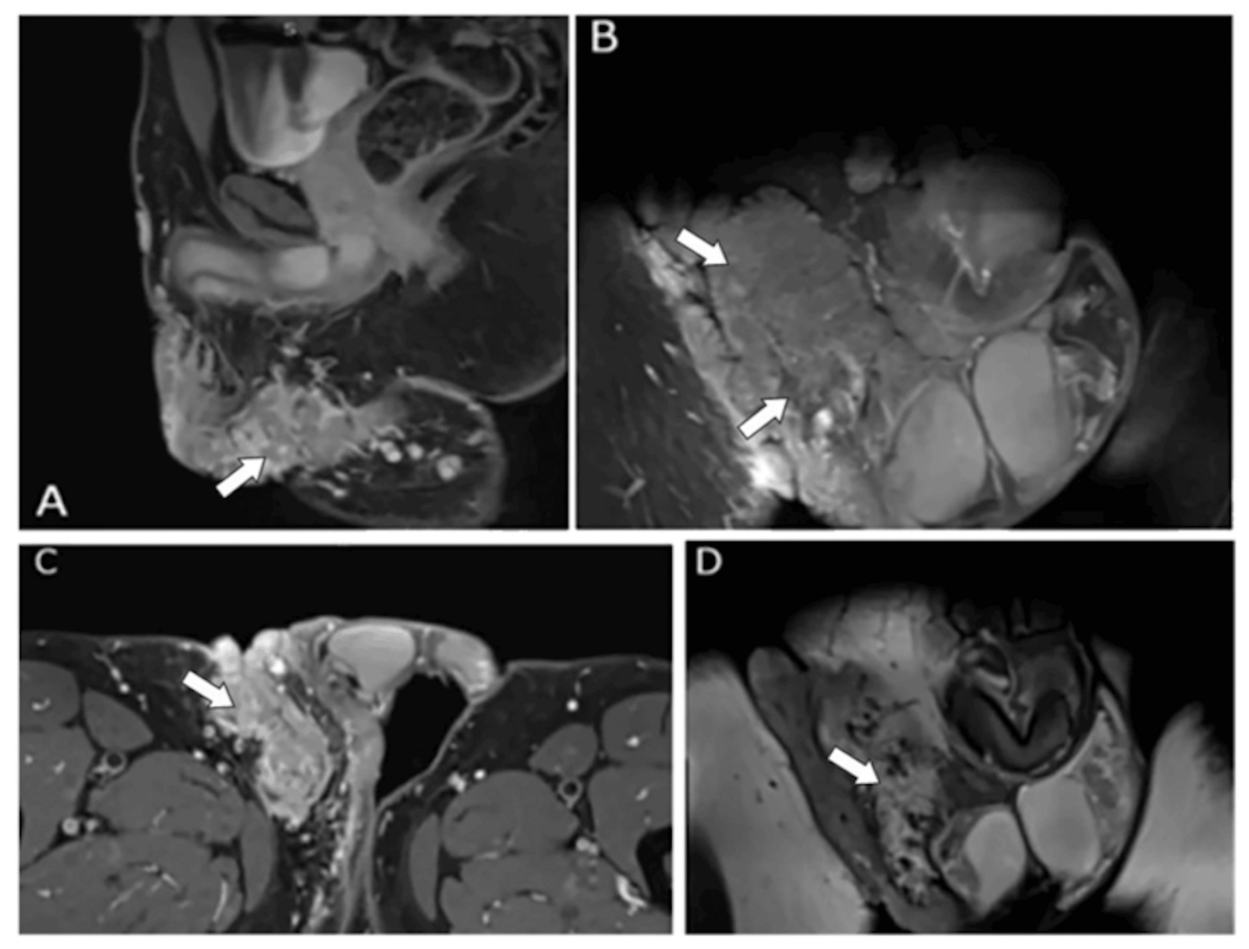
MRI scans. (A) Sagittal image. (B) Coronal image. (C) Traverse image. (D) Coronal image. Solid tissue mass anteriorly over the skin in front of the penis, along the right proximal thigh, reaching the inferomedial surface of the perineum, the right inguinal-genital region, and the skin over the right hemiscrotum. Pathologically enlarged lymph nodes are noted in the pelvic floor.

The MRI demonstrated a solid tissue mass originating from the skin and infiltrating the subcutaneous tissue of the lower anterior abdominal wall. Its upper margin was located approximately at the level of the pubic symphysis. The lesion extended anteriorly over the skin in front of the penis, continued along the right proximal thigh, reaching the inferomedial surface of the perineum, the right inguinal-genital region, and the skin over the right hemiscrotum. However, the lesion was in close contact with the tunica of the right testis. Pathologically enlarged lymph nodes were noted in the pelvic floor. One pathological lymph node, located posterior to the right external iliac vessels, just before the transition into the common femoral vessels, measured 21 × 33 mm and exerted mass effect on the right lateral wall of the urinary bladder, without any visible bladder wall involvement. In the right inguinal region, lymph nodes were visualized with a short axis of approximately 13 mm and an estimated long axis of ~3 cm (Figures 3A-3D).

Upon admission, a preoperative electrocardiogram and chest X-ray were performed, both without pathological findings. Laboratory testing revealed leukocytosis at 12.80 × 10³/μL. Biochemical and coagulation profiles were within normal range. The serological tests for human immunodeficiency virus, hepatitis A virus, hepatitis B virus, and hepatitis C virus were negative (Table 1).

**Table 1:** Laboratory findings.

Parameter	Preoperative	Postoperative	Normal range
White blood cells	12.80 × 10³/mL	12.70 × 10³/mL	4.0–10.5 × 10³/mL
Neutrophils	71.2%	79.4%	40–75%
Hematocrit	40.2%	40.2%	40–52%
Hemoglobin	13.4 g/dL	12.5 g/dL	13–17.6 g/dL
Platelets	257 × 10³/mL	224 × 10³/mL	140–450 × 10³/mL
International normalized ratio	0.98	-	0.80–1.20
Urea	30 mg/dL	20 mg/dL	10–50 mg/dL
Creatinine	0.79 mg/dL	0.75 mg/dL	0.60–1.20 mg/dL
Potassium	4.2 mmol/L	3.9 mmol/L	3.5–5.1 mmol/L
Sodium	139 mmol/L	145 mmol/L	136–145 mmol/L
Blood type and Rhesus	O (+)	-	-
Human immunodeficiency virus	Negative	-	-
Hepatitis A virus	Negative	-	-
Hepatitis B virus	Negative	-	-
Hepatitis C virus	Negative	-	-

Under general anesthesia, the patient was placed in a lithotomy position. An excision of a large lesion located at the base of the penis was performed, extending to the right hemiscrotum and the right inguinal-thigh fold. Additionally, a second lesion at the left inguinal-thigh fold was also excised. The excised lesions were sent to the laboratory for pathological analysis. Meticulous hemostasis was achieved by using bipolar diathermy. Due to the extensive skin defect, reconstruction was performed using skin flaps (Figure 1B) in collaboration with a plastic surgeon. The excision and reconstruction were performed in the same sitting, while no frozen biopsies were sent for histological analysis before the reconstruction. Layered closure was performed with intradermal suturing using 2-0 Vicryl Rapid sutures. A two-way 16-Fr Foley catheter was placed. Sterile dressings and adhesives were applied to the wound. The patient recovered uneventfully in the operating room.

On postoperative day one, laboratory tests showed mild leukocytosis with neutrophil predominance and slightly lower hemoglobin and sodium (Table 1). The patient remained afebrile, and upon removal of the dressings, there was no sign of contamination or dehiscence of the wound. New dressings were applied, and the Foley catheter was removed. No difficulty in urination was noted. After being given wound care instructions and an oral antibiotic regimen, the patient was discharged.

In the following days, given the tension of the region, the surgical edges of the wound in both inguinal regions separated superficially. The surgical wound remained free of necrotic tissues or signs indicative of infection, such as pus leaking. Besides daily wound care with normal saline, topical antibiotic and anaplastic ointment were applied. Figure 1C and Figure 1D depict the course of the wound closure in one and two weeks postoperatively, while Figure 1E demonstrates the complete closure of the surgical edges four weeks postoperatively.

**Figure 4:**
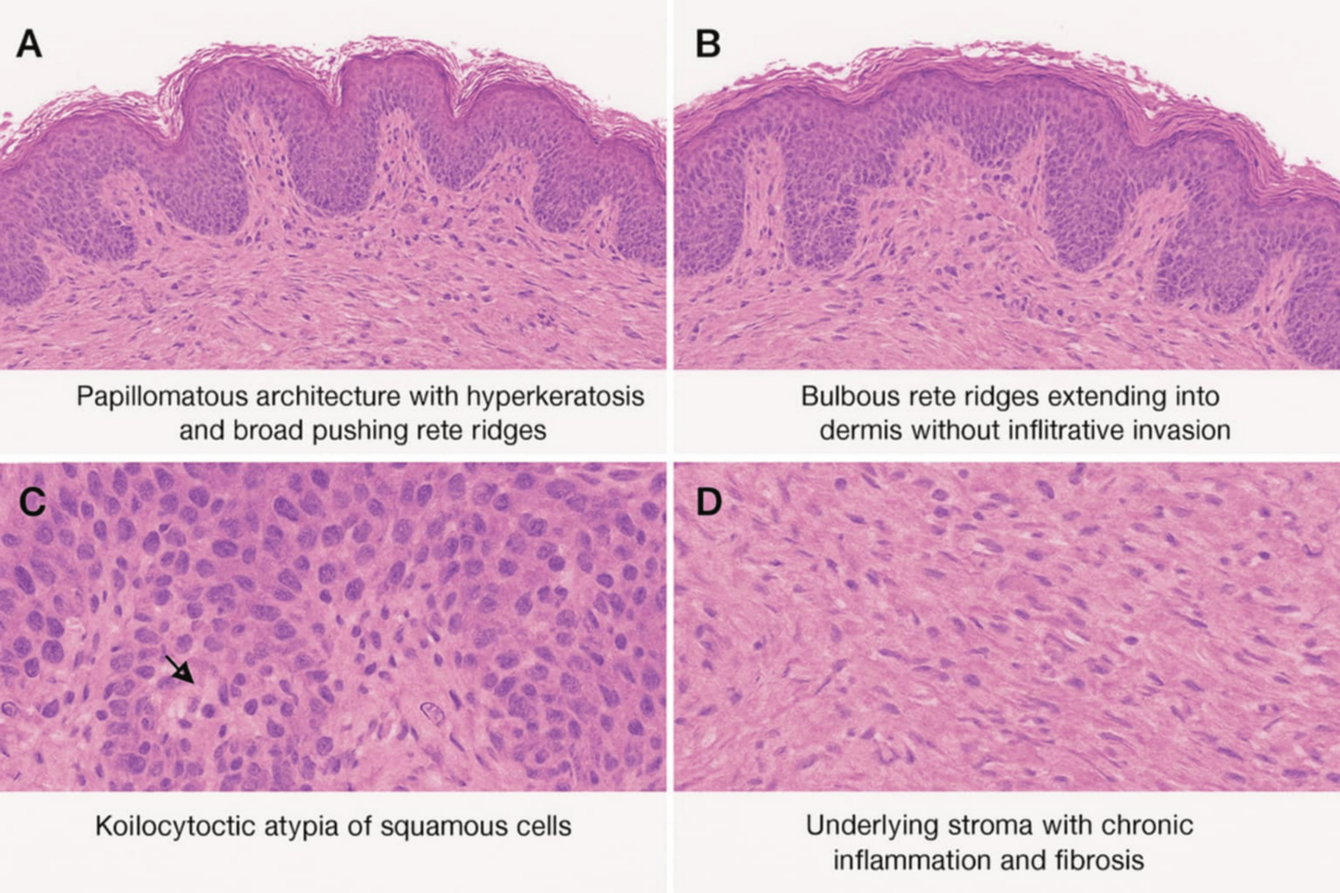
Histopathological images of the excised lesions.

The histological analysis in its gross description revealed an irregular, cauliflower-like, tan-white tissue mass measuring 7.2 × 4.6 × 3.5 cm. The surface was verrucous with focal ulceration. The cut surface was soft, fleshy, and pale tan, with areas of keratinous debris. The lesion appeared well-circumscribed. Microscopically, sections showed a markedly papillomatous and acanthotic squamous epithelium with hyperkeratosis, parakeratosis, and papillomatosis. There was extensive koilocytotic atypia of the squamous cells, characterized by perinuclear clearing and irregular nuclear contours, consistent with HPV effect. The epithelium showed pushing borders, extending into the underlying dermis and subcutaneous tissue, forming large endophytic lobules. However, there was no evidence of stromal desmoplasia or frank infiltrative invasion characteristic of conventional SCC. Numerous broad rete ridges extended downward with bulbous edges, compressing the surrounding tissue but maintaining a blunt, non-infiltrative margin. There was no lymphovascular or perineural invasion identified. Focal areas exhibited chronic inflammation, fibrosis, and reactive vascular changes in the stroma. No necrosis was noted. Immunochemistry revealed p16 patchy positivity, suggestive of low-risk HPV infection and not consistent with high-risk, HPV-driven dysplasia. Additionally, Ki-67 was positive, indicating an increased proliferation index in the basal layer only (Figures 4A-4D).

During the patient’s follow-up, no recurrence was recorded 12 months after the excision of the lesions.

## Discussion

BLT, or GCA, is a rare, exophytic, anogenital lesion characterized by aggressive local invasion despite its histologically benign appearance, an observation repeatedly confirmed in the literature [11]. This discrepancy between clinical behavior and benign histopathology underlines the importance of early recognition and intervention to mitigate potential morbidity [12]. Although malignant transformation to SCC has been reported, occurring in up to 56% of BLT cases in some analyses [12,13], our patient’s tumor remained histologically benign. This aligns with documented cases in which prompt or aggressive excision revealed no malignant foci [12,14]. Such outcomes emphasize that while vigilance is necessary, not all BLTs progress to malignancy.

The foundational treatment for BLT is radical surgical excision with wide margins, widely acknowledged as the gold standard [2,15], with locally limited lesions requiring only excision with open wound granulation [2]. In more extensive lesions, reconstructive surgery following the resection (using skin grafts such as local, scrotal flaps) is necessary due to the extensive soft-tissue deficit. Abdominoperineal resection is reserved only for cases with anorectal invasion or malignant transformation. Preserving function is challenging, mainly because of the high frequency of anal stenosis and scar formation [16]. In line with this, our case underwent complete excision, addressing both tumor clearance and the extensive soft tissue defect.

Other therapies have been explored, particularly in patients who are poor surgical candidates or present with recurrent/multifocal disease. These include topical and systemic agents such as podophyllin, intralesional or topical 5-fluorouracil, imiquimod, interferon therapy, and photodynamic therapy. Laser ablation (CO₂) and cryotherapy have also been used with variable results. However, the efficacy of these modalities remains inconsistent, with high recurrence rates when used alone [17].

Neoadjuvant therapies, such as radiotherapy, chemotherapy, and immunotherapy (e.g., interferon), are mostly reserved for recurrent cases, as they have otherwise shown limited efficacy in eliminating the disease [2]. Mortality rate is 20-30%, with local invasion and malignant transformation as the main factors contributing to death [2,12]. Current consensus supports these approaches only as adjuvant or salvage therapies, with radical surgery remaining the definitive treatment [2].

To reconstruct the resultant defect, we utilized flap-based surgery, a strategy supported by several PubMed-indexed reports. In an 11-patient series with perianal BLT, wide excision followed by V-Y flap reconstruction achieved high healing rates with minimal complications and no local recurrences over long follow-up durations [18]. Another case of anorectal BLT treated with bilateral gluteal musculocutaneous V-Y advancement flaps reported excellent long-term functional and cosmetic outcomes, with no recurrence after six years [19]. Additional cases, including reconstruction with bilateral gluteal fasciocutaneous V-Y advancement flaps, underscore that flap repair effectively addresses large perianal defects post-BLT excision [20].

Emerging literature continues to reinforce the importance of multidisciplinary planning, combining surgical oncology and reconstructive expertise, to preserve function and quality of life [16]. Timely surgery, coupled with tailored reconstruction, as in our case, supports rapid healing and maintenance of genital or anorectal function.

The histological findings in this case are consistent with BLT, showing a large verrucous mass with papillomatosis, acanthosis, and koilocytotic atypia indicative of HPV effect. The presence of broad, endophytic rete ridges with pushing but non-infiltrative borders distinguishes BLT from conventional SCC, as no stromal desmoplasia, necrosis, or vascular/perineural invasion was identified. Patchy p16 expression and basal Ki-67 proliferation suggest association with low-risk HPV infection rather than high-risk oncogenic types. These features underscore the paradoxical nature of BLT as a benign but locally aggressive lesion with a significant risk of recurrence and potential malignant transformation, emphasizing the importance of complete excision and long-term surveillance [12,21].

Given the high recurrence rates (up to ~67%) and potential (albeit absent in this case) for malignant transformation, long-term surveillance is essential, even when initial histology shows benign findings [2,12]. In our case, a relatively short surveillance period of one year postoperatively should be acknowledged. An ideal follow-up plan, which will be implemented for this patient, is an outpatient visit every 6-12 months for a total of three years postoperatively.

## Conclusions

BLT is a rare but clinically significant anogenital lesion due to its paradoxical behavior of histological benignity and aggressive local invasion, with a considerable risk of recurrence and malignant transformation. Radical surgical excision with wide margins remains the gold standard for management, often necessitating reconstructive procedures to restore function and minimize morbidity. Our case highlights the importance of early recognition, multidisciplinary surgical planning, and vigilant long-term follow-up to reduce recurrence and ensure favorable oncologic and functional outcomes.

## References

[1] Müdüroğlu M, Güllüoğlu YB, Taşlıpınar M (2024). An extraordinary case of Buschke-Lowenstein tumor: multiple localization, malignant transformation, and clinical insights—a case presentation and literature review. Afr J Urol.

[2] Nieves-Condoy JF, Acuña-Pinzón CL, Chavarría-Chavira JL, Hinojosa-Ugarte D, Zúñiga-Vázquez LA (2021). Giant condyloma acuminata (Buschke-Lowenstein tumor): review of an unusual disease and difficult to manage. Infect Dis Obstet Gynecol.

[3] Boda D, Cutoiu A, Bratu D, Bejinariu N, Crutescu R (2022). Buschke-Löwenstein tumors: a series of 7 case reports. Exp Ther Med.

[4] Ahsaini M, Tahiri Y, Tazi MF (2013). Verrucous carcinoma arising in an extended giant condyloma acuminatum (Buschke-Löwenstein tumor): a case report and review of the literature. J Med Case Rep.

[5] Buschke A, Löwenstein L (1925). Über carcinomähnliche condylomata acuminata des penis. Klin Wochensch.

[6] Chen MT, Ong F, Phan-Thien KC (2025). Buschke-Löwenstein tumour: surgical management and literature review of an unusual disease. Cureus.

[7] Zhang D, Gonzalez RS, Feely M (2020). Clinicopathologic features of Buschke-Löwenstein tumor: a multi-institutional analysis of 38 cases. Virchows Arch.

[8] Lynde C, Vender R, Bourcier M, Bhatia N (2013). Clinical features of external genital warts. J Cutan Med Surg.

[9] Kabila B, Imrani K, Kaddouri S, Boujida I, Zouaidia F, Billah NM, Nassar I (2024). Buschke-Lowenstein tumor in the penis and anorectal region: case report. Oxf Med Case Reports.

[10] Ledouble V, Sclafani F, Hendlisz A, Gomez Galdon M, Liberale G (2021). Buschke-Löwenstein tumor in a human immunodeficiency virus-positive patient: a case report and short literature review. Acta Gastroenterol Belg.

[11] Irshad U, Puckett Y (2025). Giant Condylomata Acuminata of Buschke and Lowenstein. https://www.ncbi.nlm.nih.gov/books/NBK560714/.

[12] Grosu-Bularda A, Hariga CS, Dumitru CS (2024). Clinicopathological findings and comprehensive review of Buschke-Lowenstein tumors based on a case study. J Pers Med.

[13] Purzycka-Bohdan D, Nowicki RJ, Herms F, Casanova JL, Fouéré S, Béziat V (2022). The pathogenesis of giant condyloma acuminatum (Buschke-Lowenstein tumor): an overview. Int J Mol Sci.

[14] Monteiro D, Varejão AM, Sampaio J, Rodrigues M (2022). Vulvar condyloma of Buschke and Löwenstein: an unusual tumour in developed countries. BMJ Case Rep.

[15] Mihailov R, Tatu AL, Niculet E (2023). Surgical management of perianal giant condyloma acuminatum of Buschke and Löwenstein: case presentation. Life (Basel).

[16] Barrow BE, Ansah KO, Naga HI, Sarantos N, Inman BA, Peterson AC, Erdmann D (2025). Giant condyloma acuminatum: a review of reconstructive options. Ann Plast Surg.

[17] Fanget F, Pasquer A, Djeudji F, Chabanon J, Barth X (2017). Should the surgical management of Buschke-Lowenstein tumors be aggressive? About 10 cases. Dig Surg.

[18] Yildiz A, Leventoglu S, Yildiz A, Inan A, Mentes BB (2023). Radical surgical management of perianal giant condyloma acuminatum of Buschke and Löwenstein: long-term results of 11 cases. Ann Coloproctol.

[19] Ulas M, Bostanci EB, Teke Z, Karaman K, Ercan M, Sakaogullari Z, Akoglu M (2013). Giant anorectal condyloma acuminatum of buschke-lowenstein: successful plastic reconstruction with bilateral gluteal musculocutaneous v-y advancement flap. Indian J Surg.

[20] Gürbulak EK, Akgün İE, Ömeroğlu S, Öz A (2015). Giant perianal condyloma acuminatum: reconstruction with bilateral gluteal fasciocutaneous V-Y advancement flap. Ulus Cerrahi Derg.

[21] Saikaly LE, Saikaly SK, Norman R (2020). Buschke-Loewenstein tumor: a case report and advocacy for human papillomavirus vaccination. Cureus.

